# Renal function assessment in older people: comparative analysis of estimation equation with serum creatinine

**DOI:** 10.3389/fmed.2024.1477500

**Published:** 2024-12-04

**Authors:** Stefania Peruzzo, Silvia Ottaviani, Luca Tagliafico, Mariya Muzyka, Marta Ponzano, Cristina Marelli, Alessio Signori, Alessio Nencioni, Fiammetta Monacelli

**Affiliations:** ^1^Department of Internal Medicine and Medical Specialties, University of Genoa, Genoa, Italy; ^2^Ospedale Policlinico San Martino IRCCS, Genoa, Italy; ^3^Department of Health Sciences – Section of Biostatistics, University of Genoa, Genoa, Italy

**Keywords:** eGFR equations, older adults, chronic kidney disease, creatinine, BIS1, CKD-EPI, FAS, MDRD

## Abstract

**Introduction:**

Age-related changes occurring in the kidney can lead to a reduction in Glomerular Filtration Rate (GFR); especially in older adults with multimorbidity and/or frailty, an accurate evaluation of kidney function is critical. For the estimation of GFR in patients over 70 years, CKD-EPI (Chronic Kidney Disease Epidemiology Collaboration) is often used. However, validated equations exist for old-age populations like BIS1 (Berlin Initiative Study 1) and FAS (Full Age Spectrum). Here we aimed to compare the performance of CKD-EPI, MDRD (Modification of Diet in Renal Disease), BIS1, and FAS in assessing eGFR in a population of patients over 70, to evaluate which equations show the most accurate performance in our setting.

**Materials and methods:**

A total of 499 older adults were consecutively recruited in the Orthogeriatric ward and Oncogeriatrics clinic of IRCCS Polyclinic San Martino in Genoa Italy. eGFR was calculated using CKD-EPI, MDRD, BIS1, and FAS, calculating mean, median, standard deviation, and interquartile range. Bland–Altman graphs were used to evaluate how each equation performs with respect to the others and the concordance of the attribution of the KDIGO CKD stage was performed with Cohen’s K constant and chi-squared test.

**Results:**

Patients’ mean age was 82.6 years (± 7.44), and the mean creatinine value was 0.97 (± 0.71) mg/dl. The mean value of eGFR was 70 mL/min with CKD-EPI (± 20.6) and MDRD (± 25.7), 57 mL/min with BIS1 (± 16.7) and FAS (± 19.0), respectively. BIS1 and FAS estimated lower eGFR values than CKD-EPI and MDRD. As age increases, a steady decrease in filtrate value is observed with BIS1 and FAS. MDRD and CDK-EPI do not show the same trend. The performance of the equations at a fixed eGFR value of 30 mL/min is more linear for BIS1 and FAS compared with CKD-EPI and MDRD. Upon evaluation with chi-square, the attribution of KDIGO stage was statistically different among the various equations.

**Discussion:**

An appropriate assessment of renal function is of key clinical relevance to prevent adverse outcomes and risk of drug accumulation in older adults. Our study originally showed that in persons aged more than 70 years old BIS1 is the most accurate formula in calculating eGFR values when only serum creatinine is available.

## Introduction

Renal aging is a multifaceted process characterized by anatomical and functional changes (1–4), that accumulate over a lifetime, resulting in a progressive loss of renal function (5). This age-related decline has significant clinical implications in older adults, causing increased overall morbidity (6), mortality (7), disability, and reduced quality of life (8). Moreover, this progressive loss in renal function may lead to drug overdosage (9), adverse drug reactions (10, 11), especially in patients with polypharmacy (12) and, if not properly assessed, to the progression to end-stage renal disease.

Estimated Glomerular Filtration Rate (eGFR) equations are routinely used to assess kidney function; simple creatinine-based equations are the most commonly used in clinical practice. However, although such methods are widely applied, the presence of age-related muscle loss and reduced protein intake in older adults, including clinical conditions such as sarcopenia, malnutrition, multimorbidity, and frailty (1), may cause an overestimation of eGFR, making creatinine an unreliable marker of renal function. To overcome this pitfall, a series of GFR equations have been developed to suit the timely detection and management of Chronic Kidney Disease (CKD) in older adults (13).

The Modification of Diet in Renal Disease (MDRD) (14) and the Chronic Kidney Disease Epidemiology Collaboration (CKD-EPI) (15) equations have been widely used for renal function estimation, despite the underrepresentation of older adults in the original population studies used for their validation process. To address this gap, the Berlin Initiative Study Equation (BIS1) (16) was developed and specifically validated for older adults, including those over the age of 85 (*oldest old*). Namely, the BIS1 formula has been developed in different population cohorts, from individuals with normal renal function to those with CKD and renal transplant recipients, positioning the BIS1 formula as a valuable tool for estimating renal function in a broad spectrum of populations. Similarly, the Full Age Spectrum (FAS) (17) was designed with the goal of adapting to the whole life span of an individual, from childhood to old age; in order to accomplish that, a different equation is used based on the age of the subject: Schwarz (for children), CKD-EPI (for adults under 70), BIS1 (for adults over 70).

Existing evidence has pointed out that BIS1 and FAS, compared with CKD-EPI had a better performance in older adults (18). In line with that, Koppe et al. compared the reliability of MDRD, CKD-EPI, and BIS1 equations in patients over 70 years of age, using gold standard measurements of renal clearance based on inulin and suggesting that the BIS1 equations showed the smallest interquartile range, less variability, with higher precision and the highest coefficient of agreement (19). Moreover, Oscanoa et al. (20) evaluated the performance of eGFR equations across 1,295 studies (16 were included in the metanalysis), underscoring that BIS1 was the most accurate eGFR estimate in older adults, particularly in those with GFR values ≥60 mL/min/1.73m (2).

Recently, Beridze et al. (21) have assessed the concordance between five different equations such as (MDRD, CKD-EPI, Revised Lund-Malmö [RLM], BIS1, and European Kidney Function Consortium [EKFC]) in a study of 3,094 older adults (63.7% female), with a median age of 72 years. The results underscored the highest concordance between RLM and EKFC, while MDRD and CKD-EPI yielded higher eGFR estimates compared to the other equations, concluding that eGFR equations were not interchangeable and that further validation studies against measured GFR are highly warranted. These findings are in contrast with those of Torreggiani et al. (22), who showed, using CKD-EPI as a reference, that the use of different renal formulae did not substantially change the overall eGFR estimates.

So far there is scant evidence including age-specific equations to estimate renal function in older adults and investigating their agreement to appropriately depict CKD in real-world populations. Additionally, the association between eGFR equations and health outcomes in older adults with multimorbidity and frailty remains understudied. In line with that, Montesanto et al. (23) underscored the presence of an U-shape relationship between eGFR values and mortality in the oldest old, suggesting that the availability of an accurate assessment of eGFR, particularly in those patients with multimorbidity or frailty, could hold a significant prognostic value.

Based on this background, the present study is aimed at assessing the concordance among MDRD, CKD-EPI, BIS1, and FAS eGFR estimations, to investigate to what extent CKD may be staged interchangeably by these equations in a very old-age population sample.

## Materials and methods

We conducted a retrospective cross-sectional study at the Orthogeriatric Unit and outpatient Oncogeriatric clinic of IRCCS Polyclinic San Martino, Genoa, Italy.

The exclusion criteria were age above 65 years old, lack of consensus to participate in the study, the absence of any creatinine values.

Data were collected from January to December 2021 and included age, sex, and serum creatinine (after overnight fasting). For patients admitted to the Orthogeriatric ward blood sampling was conducted during the first day of hospitalization.

The IRCCS Polyclinic San Martino general laboratory used the standardized enzymatic method to measure creatinine levels.

This study was approved by the IRB (CERA N 2024–54 12/06/2024), University of Genoa, Italy.

Estimated Glomerular Filtration Rate Equations: eGFR was estimated by MDRD (14), CKD-EPI (15), BIS1 (16), and FAS (17) as follows.

MDRD:


$$\begin{array}{l} \mathrm{eGFR}=175*{S_{cr}}^{-1.184}*\mathrm{ag}e^{-0.203}*0.742\;\left(\mathrm{if female}\right)*\\ 1.212\;\left(\mathrm{if black}\right) \end{array}$$

CKD-EPI:


$$\begin{array}{l} \mathrm{eGFR}=141*\min{\left(S_{cr},\kappa \right)}^{\alpha }*\max{\left(S_{cr},\kappa \right)}^{1.209}*\\ 0.993^{\mathrm{age}}*1.018\;\left(\mathrm{if female}\right) \end{array}$$

where *κ* = 0.7 for women and 0.9 for men; *α* = −0.329 for women and − 0.411 for women; min indicates the minimum of SCr/κ or 1; and max indicates the maximum of SCr/κ or 1.

BIS1:


$$\mathrm{eGFR}=3.736*{S_{cr}}^{-0.87}*\mathrm{ag}e^{-0.95}*0.82\;\left(\mathrm{if female}\right)$$

FAS:


$$\mathrm{eGFR}=107.3/\left[\frac{S_{cr}}{Q}*0.988^{\mathrm{age}-40\left(\mathrm{when}\;\mathrm{age}\;\mathrm{is}>40\right)}\right]$$

Q = 0.70 for female, 0.90 for male;

For each equation mean, median, standard deviation (SD) and interquartile range (IQR) were calculated. Graphical representations of the distribution of eGFR values were displayed using histograms. To assess the agreement between the eGFR values, Bland–Altman-type scatter plots were built.

Student’s T-test was used to preliminary evaluate the distribution of eGFR values in males and females and a linear regression model was built with sex as a predictor to assess any sex difference in the examined population.

Patients were grouped into eGFR categories based on KDIGO guidelines for CKD (24). Based on this categorization, the concordance in the attribution of CKD between the equations stage was evaluated using a chi-squared test. Subsequently, the concordance with KDIGO renal failure staging was performed using Cohen’s constant K (25).

Statistical analysis was conducted using Rstudio (version 2023.06.0) with a statistical significance set at two-sided *α* less than 0.05 and Excel.

## Results

499 consecutive patients were recruited between January and August 2021. The mean age was 82.6 years (±7.44), with a predominance of females (74%). The mean creatinine value was 0.97 mg/dL (± 0.71).

**Figure 1:**
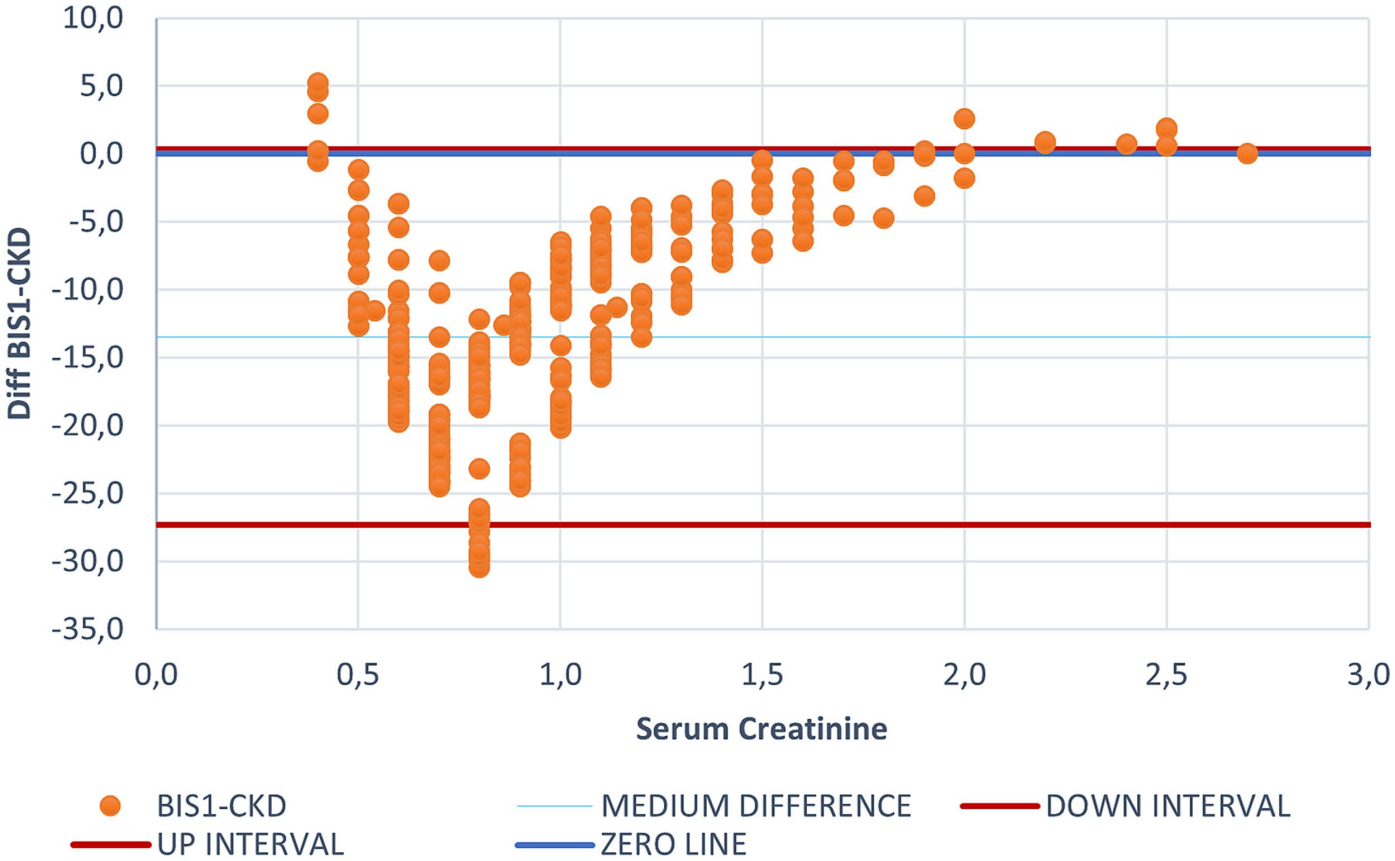
Bland Altman graph plotting the difference between CKD-EPI and BIS1 for each creatinine value. This figure illustrates the differing behaviors of BIS1 and CKD-EPI, with each dot representing an individual patient. The blue line indicates the zero threshold, while the two red lines represent the upper and lower confidence intervals. It is to note that most values fall below the zero threshold, suggesting that BIS1 tends to yield lower eGFR values compared to CKD-EPI.

As shown in Table 1, BIS1 (SD 16.7) and FAS (SD 19.7) showed the least dispersion in eGFR values. Figure 1 illustrates the difference among eGFR values using BIS1 and CDK-EPI (the latter used as a reference category).

**Table 1:** Summary of eGFR estimates.

	CKD-EPI	MDRD	BIS1	FAS
Mean	70.5	70.9	57.0	57.6
Median	72.6	68.7	56.3	56.6
SD	20.6	25.7	16.7	19.7
IQR	29.3	28.3	20.5	23.1

CKD-EPI, Chronic Kidney Disease Epidemiology Collaboration; MDRD, Modification of Diet in Renal Disease; BIS1, Berlin Initiative Study Equation; FAS, Full Age Spectrum; SD, Standard Deviation; IQR, Inter-Quartile Range.

Bland–Altman plots showed that the eGFR value estimated by CKD-EPI was generally higher than that estimated by BIS1, suggesting that the CDK-EPI tends to overestimate eGFR. Only for creatinine values below 0.4 and above 1.7 mg/dL CKD-EPI provided lower eGFR estimates compared to BIS1. The median difference between eGFR calculated with BIS1 and with CKD-EPI was 13.49 mL/min (IQR 9.85 mL/min). Within creatinine values below 0.4 mg/dL and above 1.7 mg/dL all the equations seemed to be less accurate in estimating renal function.

Being FAS formula derived from BIS1, plotting the difference between FAS and CKD-EPI (Supplementary Figure S1) displayed a similar trend compared to that observed with BIS1.

FAS yielded a higher eGFR estimate than CKD-EPI for creatinine values below 0.4 and above 1.7 mg/dL. Notably, FAS showed no values above the zero line for creatinine values suggestive of renal failure.

**Figure 2:**
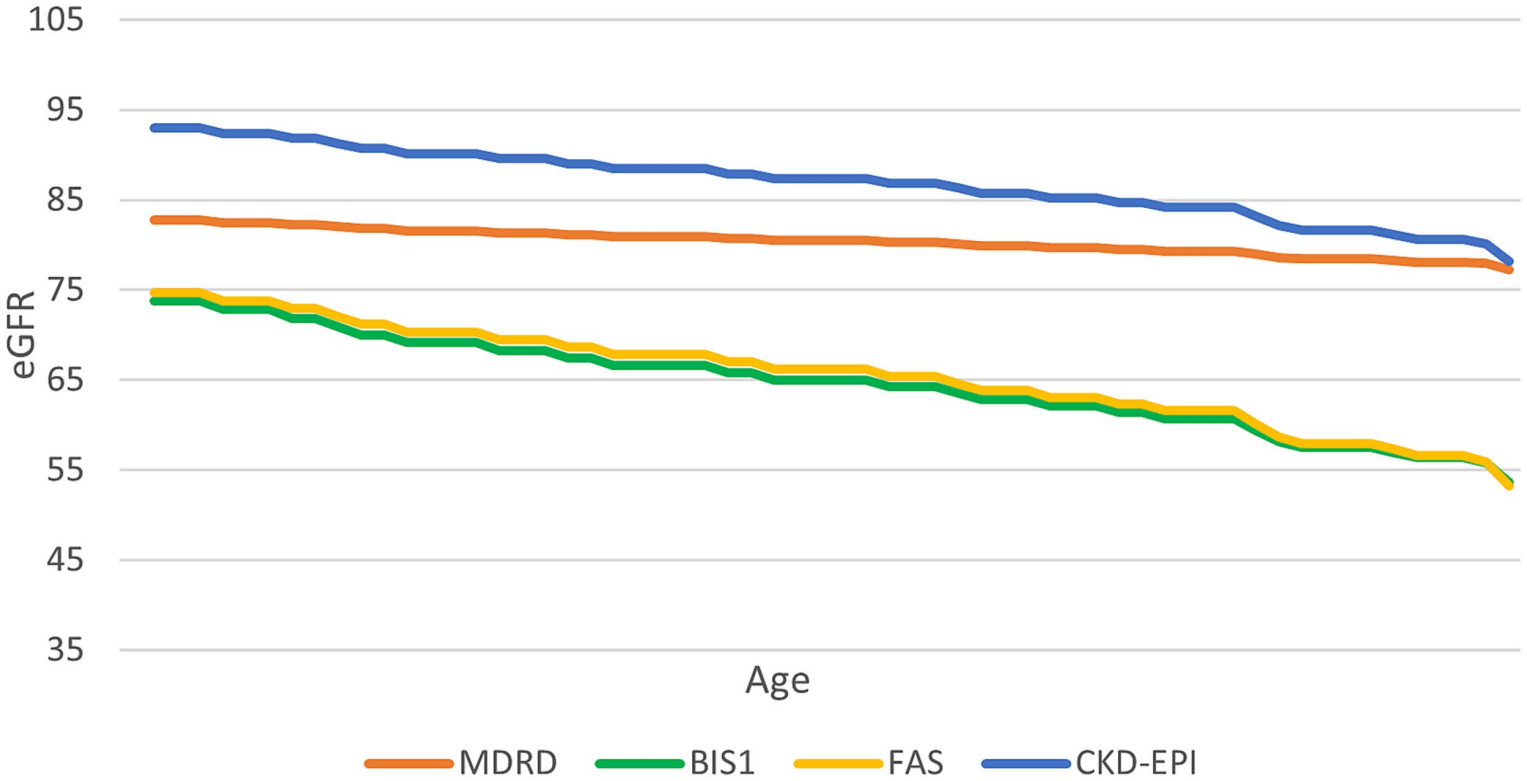
Trend of the performance of different equations at a serum creatinine value of 0.7 mg/mL. This graph illustrates how different equations perform as age increases, with a constant creatinine value of 0.7 mg/dL.

To further assess the variation in the eGFR formula according to aging, we fixed a creatinine value of 0.7 mg/dL and examined the change in eGFR estimates as the age increased (Figure 2). CKD-EPI consistently yielded higher eGFR values compared to BIS1 and FAS for this specific creatinine level.

**Figure 3:**
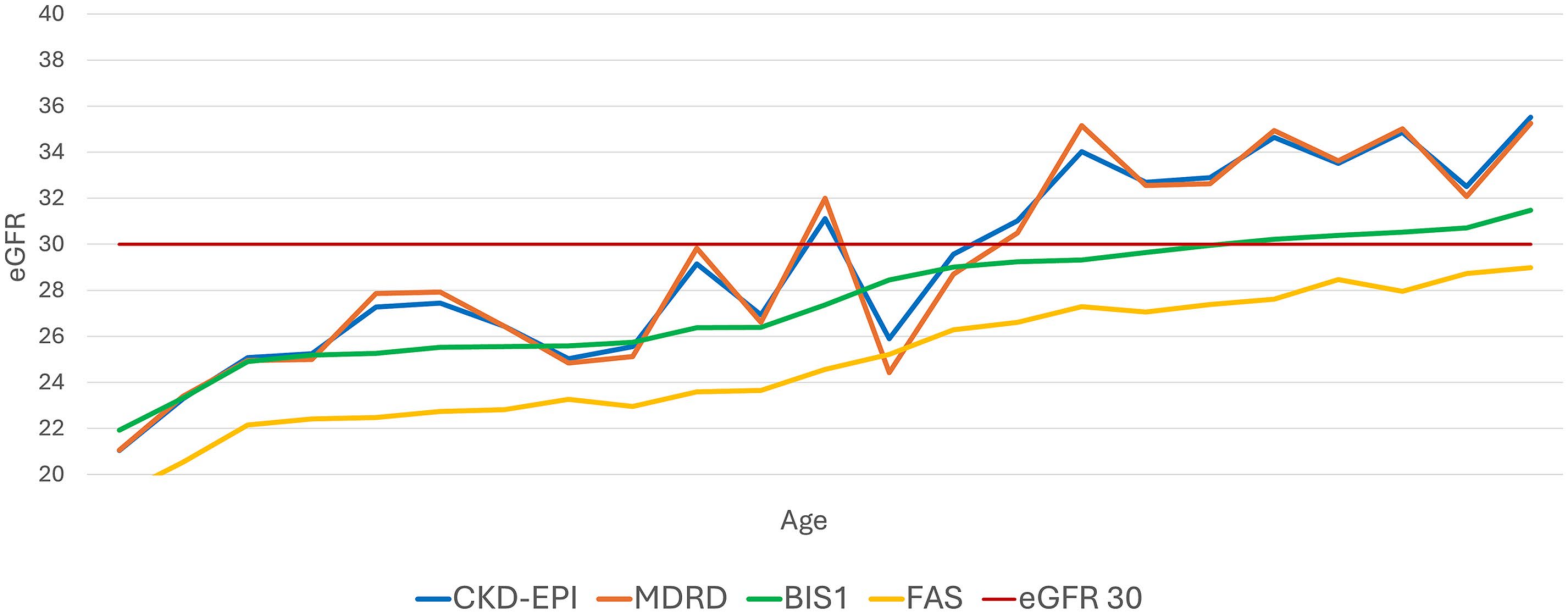
Trend of the performance of different equations with an evidenced fixed value of eGFR 30 mL/min/1.73 m^2^. This graph illustrates how the various equations relate to an eGFR value of 30 mL/min as age increases.

Subsequently, we set a constant eGFR value of 30 mL/min/1.73m^2^ and analyzed the fluctuation of creatinine levels as age increased (Figure 3). CKD-EPI and MDRD showed a less linear trend compared to BIS1 and FAS, which displayed a steadier increase.

The stratification for sex showed a significant difference in males (mean value of creatinine was 1.24 mg/dL), versus 0.88 mg/dL in females (*p*-value of 0.001).

In light of the presence of such sex difference, the comparative analysis of the performance of BIS1 and CKD-EPI showed a statistical trend (*p*-value of 0.054); a linear regression model, adjusted for age, supported the significance of this finding (*β* −1.55, 95% CI −2.94 – −0.16, p-value 0.03). The analysis was replicated using FAS formula (*vs* CKD-EPI), that did not reveal any significant sex difference.

The categorization of renal function using the KDIGO classification was performed using each equation (Table 2). A chi-squared test was used to evaluate the performance of each equation in the attribution to the respective stage. We obtained a *p* < 0.001 with *χ* of 223 and 95% threshold of 25. Notably, in our sample, the attribution of stage G3b (eGFR between 30 and 44 mL/min) was significantly different across the equations: BIS1 and FAS showed a two-fold higher agreement in estimating stage G3b than MDRD and CKD-EPI.

**Table 2:** Attribution CKD stage, according to KDIGO Guidelines, for each equation.

	CKD-EPI	MDRD	BIS1	FAS
G1 (>90)	90	118	17	28
G2 (60–89)	265	205	185	178
G3a (45–59)	82	110	188	175
G3b (30–44)	38	42	80	83
G4 (15–29)	20	20	26	31
G5 (<15)	4	4	3	4

The concordance analysis yielded Cohen’s kappa ranging from a minimum of 0.75 (good agreement) between CKD-EPI and BIS1, to a maximum of 0.97 (excellent agreement) between FAS and BIS1 (see Supplementary Figure S2).

## Discussion

The estimation of renal function in older adults poses several challenges due to age-related physiological changes, multimorbidity, frailty and medication use. It is noteworthy that an imprecise evaluation of renal function can lead to inappropriate drug prescribing and overdosing, particularly with antibiotics (26–28), anti-diabetic drugs (29), or anticoagulants (30–32), which increases the risk of iatrogenesis. Therefore, it is crucial to tailor the estimation of renal equations in such vulnerable populations.

In the present study BIS1 and FAS showed the least dispersion in eGFR values, an excellent agreement, and a linear age-related increase as creatinine worsens, especially in the presence of an eGFR value below 30 mL/min/1.73m^2^. As such, our findings originally support the notion that BIS1 and FAS formulas appropriately estimate eGFR when compared to the other examined equations, which conversely displayed a consistent overestimation of eGFR.

Notably, BIS1 and FAS showed the highest concordance in the attribution of renal failure KDIGO staging, further supporting their clinical implementation in old-age patients. Moreover, based on our findings, BIS 1 and FAS allowed for the categorization of KDIGO staging, with stage G3 being the most prevalent. On the contrary, using CKD-EPI and MDRD, stage G2 was found to be the most prevalent, suggesting that inaccurate CKD staging may have potentially unfavorable clinical implications and mortality.

Ma et al. (18) in a meta-analysis demonstrated that BIS1 and FAS performed a more accurate estimation of eGFR in older adults, also suggesting that the inaccuracy of creatinine measurements might be mitigated by using equations that incorporate cystatin C, such as CKD-EPIcr-cys or BIS2 (33–36). Similarly, Xia et al. (37), underscored that BIS1 and FAS outperformed CKD-EPI in estimating renal function, especially with eGFR values under 60 mL/min/1.73m^2^.

Paparazzo et al. (38) showed that CKD-EPI, BIS1, and FAS equations cannot be considered interchangeable, suggesting that CKD-EPI tends to overestimate eGFR values and that BIS1 and FAS show better prognostic accuracy, implying a relevant impact on mortality in older frail nursing home residents. These findings are in line with those of Beridze et al. (21), which also demonstrated the best prognostic accuracy for BIS in predicting long-term mortality.

It could be hypothesized that BIS1 and FAS’s higher accuracy in estimating eGFR may lie in accounting for age-related physiological changes and muscle mass. Indeed, the muscular mass displays an age-related decline (38, 39), implying that creatinine values within the normal range of 0.7 mg/dL may be a sign of renal failure (40, 41) in older patients. In line with that, Corsonello et al. (42) pointed out the importance of incorporating muscle mass in the estimation of eGFR, as it may be a key relevant source of discrepancy, accounting for almost 11% of the difference between eGFR values estimated with CKD-EPI and BIS1.

Our findings originally showed a sex stratification in the performance of the BIS1 and CKD-EPI formulas, with men exhibiting a greater discrepancy (43, 44). In our sample males displayed higher creatinine values, although male sex being underrepresented poses a limitation for a correct understanding. It could be hypothesized that sarcopenia may account for such discrepancies (45), although the lack of inclusion of clinical variables prevents us from making any conclusive remark.

Properly assessing renal function can also exert an epidemiological impact on CKD: Ebert et al. (43–44) showed that the incidence, clinical presentation, and outcomes of CKD can widely vary depending on which eGFR formula is used. Similarly, Liu et al. (45) pointed out that the application of the same formulas and the same eGFR threshold for each age range can lead to an overestimation of renal failure diagnosis, especially in persons aged 70 years and more.

It could be argued that eGFR thresholds for defining CKD should be age-specific although up to date no clinical guideline has yet adopted any age-specific stratification for CKD. However, it is well accepted that eGFR formulas display the best performance in populations that are similar to those in which the validation process took place. Starting from this assumption, it should not come as a surprise that BIS1 and FAS formulas demonstrated better mutual concordance when compared to MDRD and CKD-EPI.

Moreover, according to our findings, when creatinine values are outside the reference range (below 0.5 or above 1.7 mg/dL), all the estimated equations (MDRD, CKD-EPI, BIS1, and FAS) fail to provide accurate estimations of eGFR, posing further clinical challenges. So far, all the equations derived from population-based normative data with a Gaussian distribution of values which is poorly established in older populations, leading to increased difficulty in differentiating whether these outside ranges may be signs of disease, the extent of renal failure severity, or related to normal renal aging.

Furthermore, the U-shaped distribution of our results is similar to that reported by Montesanto et al. (23), who showed an association between mortality and eGFR, with an increase in the former according to extreme values of the latter.

Although the preliminary nature of the study, BIS1 and FAS equations provide similar eGFR values and classification, showing that renal function and CKD cannot be staged interchangeably with other equations in a distinguished proportion of old-age patients. Indeed, BIS1 and FAS1 seem to appropriately depict a non-negligible proportion of older adults carrying an increased risk of unfavorable outcomes due to nephrotoxicity-related complications.

The routine implementation of BIS1 and FAS equations to estimate renal function in clinical practice may bring significant clinical benefits, enabling early interventions to preserve kidney function or mitigating the progression of renal failure in older adults, particularly in the presence of frailty, multimorbidity, and polypharmacy, minimizing medication risks, through the ability to optimize tailored dosage adjustments or alternative treatments to ensure patient safety and therapeutic efficacy.

The strengths of this study include the consistent sample from a real-world geriatric hospital setting with very old age and the application of four different equations for the estimation of renal function, Furthermore, the analysis of the performance of the equations in the presence of an established cut off value eGFR (30 mL/min/1.73m^2^) for drugs deprescribing or dosage adjustment brings novelty to this field, strengthening the real world application of our findings. Moreover, the creatinine measurement at a single laboratory reduced the random variability in creatinine assessment.

In contrast to Oscanoa et al. (20), who provided a comprehensive meta-analysis on the accuracy of BIS1 versus other eGFR formulas in older adults, our study advances this work by directly comparing the performance of BIS1, CKD-EPI, MDRD, and FAS, specifically focusing on very old patients. While Oscanoa highlighted BIS1’s superior accuracy, particularly for eGFR values above 60 mL/min/1.73m^2^, we extend these findings by assessing the possible applicability of BIS1 and FAS in categorizing KDIGO stages across a sample with presumably high levels of multimorbidity and frailty. Moreover, our study includes an analysis of sex-based differences in eGFR estimates and investigates the impact of specific creatinine thresholds on formula accuracy.

On the other hand, this study has several limitations. Firstly, measured eGFR was not available, therefore, it was not possible to compare the performance of the different equations with an actual gold standard. Secondly, the presence of a single-point creatinine measurement may limit the accuracy in distinguishing between transient and chronic renal failure. It is noteworthy, in fact, that the presence of femur fracture may increase the risk of acute renal failure due to bleeding or dehydration, as well as cancer and cancer-treatments could affect renal functioning. The population selection may therefore count for a selection bias.

Moreover, the absence of patients’ frailty stratification and the lack of integration of patient’s clinical variables, including sarcopenia and/or and multimorbidity, may hamper the clinical understanding of the renal burden with aging and the correlation with frailty trajectories and clinical outcomes in such an old population. Namely, different factors may influence the sensitivity of all the examined equations such as hydration, body composition, and, in particular, sarcopenia or muscle mass may account for sources of discrepancies among the examined equations.

To address these limitations, future directions of this research will aim to fill this gap of knowledge, performing an accurate frailty stratification, based on geriatric multidimensional assessment, to explore the potential clinical impact of inaccurate renal function estimates on patients’ clinical outcomes and frailty trajectories. Furthermore, we intend to fill the present lack of longitudinal data in order to broaden the clinical perspective of the investigation, by exploring the trajectories of renal function and their intersection with those of frailty and survival.

The collection of cystatin C data to improve the accuracy of eGFR calculations, even in sarcopenic patients, will be also performed.

## Conclusion

In conclusion, the accuracy of eGFR estimation is a major geriatric concern because of its implications in terms of nephrotoxicity-related implications of multimorbidity, and polypharmacy in the presence of frailty in very old individuals. The use of validated equations, such as BIS1 and FAS could increasingly supersede the use of CKD-EPI or other formulas, capturing the complexity of the biology of aging and providing new hints in the mitigating strategies for the intertwined trajectory of renal failure and frailty in older adults.

## Data Availability

The raw data supporting the conclusions of this article will be made available by the authors, without undue reservation.
