# Supplementary figures and images for: Renal function assessment in older people: comparative analysis of estimation equation with serum creatinine
Source: Front Med (Lausanne). 2024 Dec 4;11:1477500. doi: 10.3389/fmed.2024.1477500 (PMC11652175; doi:10.3389/fmed.2024.1477500)

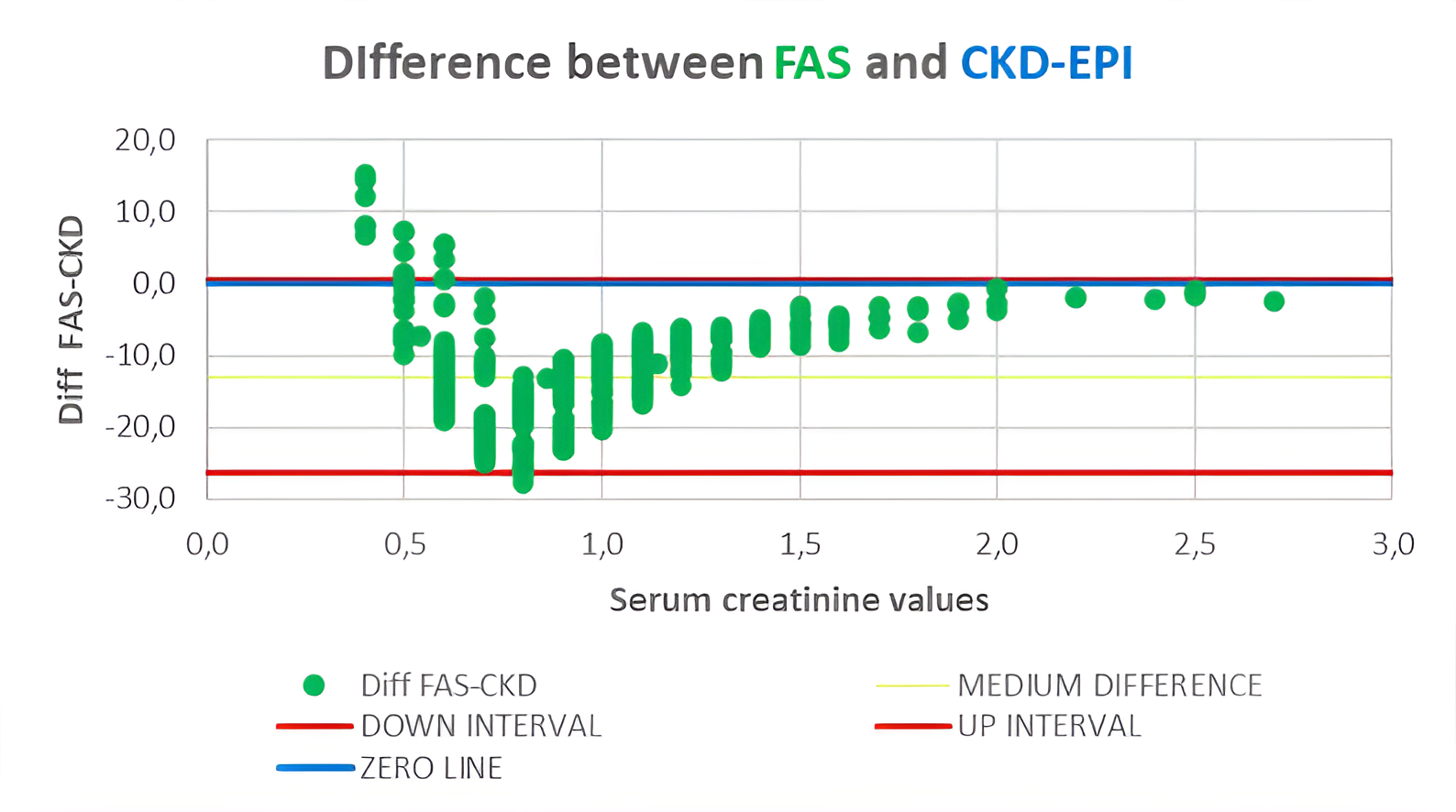

Supplement: Supplementary file 1 [file Image_1.TIF]

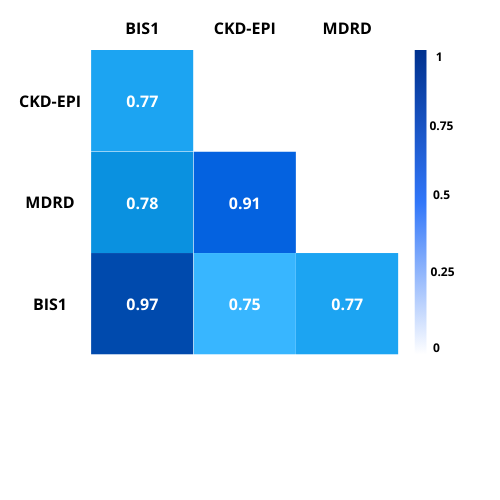

Supplement: Supplementary file 2 [file Image_2.PNG]
